# Tracking the Sleep Onset Process: An Empirical Model of Behavioral and Physiological Dynamics

**DOI:** 10.1371/journal.pcbi.1003866

**Published:** 2014-10-02

**Authors:** Michael J. Prerau, Katie E. Hartnack, Gabriel Obregon-Henao, Aaron Sampson, Margaret Merlino, Karen Gannon, Matt T. Bianchi, Jeffrey M. Ellenbogen, Patrick L. Purdon

**Affiliations:** 1 Massachusetts General Hospital, Department of Anesthesia, Critical Care, and Pain Medicine, Charlestown, Massachusetts, United States of America; 2 Massachusetts General Hospital, Department of Neurology, Massachusetts, United States of America; 3 Johns Hopkins University, Department of Neurology, Baltimore, Maryland, United States of America; University College London, United Kingdom

## Abstract

The sleep onset process (SOP) is a dynamic process correlated with a multitude of behavioral and physiological markers. A principled analysis of the SOP can serve as a foundation for answering questions of fundamental importance in basic neuroscience and sleep medicine. Unfortunately, current methods for analyzing the SOP fail to account for the overwhelming evidence that the wake/sleep transition is governed by continuous, dynamic physiological processes. Instead, current practices coarsely discretize sleep both in terms of state, where it is viewed as a binary (wake or sleep) process, and in time, where it is viewed as a single time point derived from subjectively scored stages in 30-second epochs, effectively eliminating SOP dynamics from the analysis. These methods also fail to integrate information from both behavioral and physiological data. It is thus imperative to resolve the mismatch between the physiological evidence and analysis methodologies. In this paper, we develop a statistically and physiologically principled dynamic framework and empirical SOP model, combining simultaneously-recorded physiological measurements with behavioral data from a novel breathing task requiring no arousing external sensory stimuli. We fit the model using data from healthy subjects, and estimate the instantaneous probability that a subject is awake during the SOP. The model successfully tracked physiological and behavioral dynamics for individual nights, and significantly outperformed the instantaneous transition models implicit in clinical definitions of sleep onset. Our framework also provides a principled means for cross-subject data alignment as a function of wake probability, allowing us to characterize and compare SOP dynamics across different populations. This analysis enabled us to quantitatively compare the EEG of subjects showing reduced alpha power with the remaining subjects at identical response probabilities. Thus, by incorporating both physiological and behavioral dynamics into our model framework, the dynamics of our analyses can finally match those observed during the SOP.

## Author Summary

How can we tell when someone has fallen asleep? Understanding the way we fall asleep is an important problem in sleep medicine, since sleep disorders can disrupt the process of falling asleep. In the case of insomnia, subjects may fall asleep too slowly, whereas during sleep deprivation or narcolepsy, subjects fall asleep too quickly. Current methods for tracking the wake/sleep transition are time-consuming, subjective, and simplify the sleep onset process in a way that severely limits the accuracy, power, and scope of any resulting clinical metrics. In this paper, we describe a new physiologically principled method that dynamically combines information from brainwaves, muscle activity, and a novel minimally-disruptive behavioral task, to automatically create a continuous dynamic characterization of a person's state of wakefulness. We apply this method to a cohort of healthy subjects, successfully tracking the changes in wakefulness as the subjects fall asleep. This analysis reveals and statistically quantifies a subset of subjects who still respond to behavioral stimuli even though their brain would appear to be asleep by clinical measures. By developing an automated tool to precisely track the wake/sleep transition, we can better characterize and diagnose sleep disorders, and more precisely measure the effect of sleep medications.

## Introduction

Scientists have long noted that the *sleep onset process* (SOP), the gradual transition between wakefulness and sleep, is marked by a dynamic continuum of behavioral and physiological changes [1]. Consequently, the ability to understand and provide a principled characterization of SOP dynamics in both healthy and pathological subjects is of fundamental importance for sleep medicine and basic neuroscience alike. In sleep disorders such as insomnia, which has been associated with increased morbidity and mortality [2], the time course of the wake/sleep transition may be pathologically protracted, resulting in difficulty falling asleep. In disorders of excessive sleepiness, such as narcolepsy or sleep deprivation, the wake/sleep transition occurs too rapidly, resulting in difficulty staying awake, with implications for performance and safety. While there is increasing recognition of the importance of objective sleep testing, there currently exist no quantitative metrics for clinically diagnosing insomnia, which is currently defined exclusively by patient self-report [3]. Hypersomnia, in contrast, is typically defined using a multiple sleep latency test (MSLT) [4], a labor-intensive diagnostic involving multiple nap periods, all of which must be visually scored by technicians. Given the importance of sleep onset dynamics, the ability to track the continuous dynamical properties of the SOP in a principled, automated manner could provide critical insight into the pathophysiology of these populations, aiding in both diagnosis and in treatment.

While the wake/sleep transition has been shown to be continuous and dynamic in every physiological and behavioral system studied thus far [1], current clinical and research practices unfortunately employ methodologies that essentially ignore these dynamics. This is because these analyses still rely on time-consuming, subjective discretization of the sleep process, performed by technicians who visually score the sleep time-series data in 30-second epochs according to semantically-defined sleep stages [5]. These scoring standards grossly oversimplify sleep dynamics by discretizing the data in both time (by using fixed, non-overlapping epochs) and state (by using discrete sleep stages).

Current SOP analysis discretizes the data even further by reducing the complex, dynamic interplay of neural systems and behavior to a single “point of sleep onset” using semantic criteria. Most notably, the American Academy of Sleep Medicine (AASM), defines sleep onset as the first appearance of any 30-second epoch that contains at least 15 seconds of sleep [5]. By defining a single point of sleep onset, these analyses impose a binary model on the SOP, in which in which the transition from wake to sleep occurs instantaneously at a particular moment in time. This non-physiological transition effectively removes all temporal dynamics, and thus severely limits the degree to which these methods can successfully characterize and diagnose pathologies of sleep onset. Therefore, no matter how sophisticated experimental exploration of the SOP becomes, it will always be limited by the coarse way in which dynamics are described in the analyses.

Additionally, current experimental analysis of the SOP does not consider behavioral dynamics in conjunction with the physiology, and clinical sleep medicine does not record behavior at all. A reason for this may be the fact that behavioral and physiological dynamics can evolve at different time scales, which would be difficult to characterize using traditional staging methods. It is therefore essential to employ techniques that can fully capture the dynamics of both the physiology and behavior in a principled and integrated manner.

While powerful techniques for statistical modeling of dynamic processes have been widely available for several decades [6], they have yet to be adopted in sleep medicine, nor used to characterize the multimodal dynamics of sleep. The absence of appropriate statistical paradigms for analyzing sleep dynamics is a fundamental impediment to progress in sleep medicine. Development of such statistical methods could have tremendous scientific and clinical impact. In this paper, we propose a dynamic state-space model framework for the characterization of simultaneously observed behavioral and physiological dynamics during the SOP. In doing so, we create a robust quantitative representation of SOP dynamics that can be used to more accurately and more precisely track the gradual transition from wakefulness to sleep. We use a fully Bayesian framework that facilitates flexible and rigorous statistical inferences, including comparison of SOP in different patient populations. We apply this method to data from healthy subjects, demonstrating the features of the method and providing a point of comparison for future studies of sleep pathologies.

### Defining Sleep Onset: Previous Behavioral and Physiological Metrics

There have been numerous ways in which scientists have attempted to measure behavioral and physiological dynamics during the SOP. For an in-depth look at the methods employed in the past, see Ogilvie's review paper [1], which comprehensively details the many multimodal correlates of sleep onset and the experimental strategies employed in characterizing them.

Ogilvie divides behavioral metrics of sleep onset into categories of *active* and *passive* behavioral measurement. Active metrics involve tasks with repeated externally-generated probes for wakefulness, each of which prompts the subject for a physical response via button press or verbal response. Additionally, response via cued respiration has been used for experimental and interventional behavioral paradigms [7], [8]. These active probes could include subjective queries [9]–[11], or auditory [12]–[14] and vibratory [15] stimuli. Use of a psychomotor vigilance task (PVT) derived metric [16] has also been proposed. Active methods are useful, as repeated trials yield multiple measurements of wakefulness across the entire SOP, which can be used to characterize SOP dynamics. Moreover, multiple measurements provide statistical power for descriptive and comparative data analyses. These active measurement schemes, however, have all required the use of external stimuli that are potentially arousing and can disrupt sleep [17]–[19]. It has therefore been a question of balancing the trade-off between stimulus salience and the degree to which the SOP is perturbed.

Passive behavioral methods for measuring the SOP include actigraphy [17], [20], [21], continuous pressure (dead man's switch) systems [22], or a finger tapping task [19]. Actigraphy is the most prevalent form of passive measurement, and has recently been brought to popular attention through home sleep tracking applications for mobile devices [17], [21]. Since actigraphy works under the assumption that behavioral quiescence in the absence of a task indicates sleep, it cannot distinguish between wakeful motionlessness during the SOP and actual sleep, and thus is not precise enough to describe sleep onset [1]. Passive paradigms involving the use of a “dead man's switch” or finger tapping task compress all SOP dynamics into a single data point by defining sleep onset as the moment at which behavior ceases, and thus tend to underestimate sleep latency [1].

While both active and passive behavioral metrics show general correlation with features of the SOP dynamics, neither is without issue. Therefore, an important goal is to search for a behavioral task that features multiple highly-salient trials (as with the active metrics), yet minimizes arousing external stimuli (as with the passive metrics).

As sleep is a neural process, direct observation of brain activity has been the primary means of tracking the SOP. The most obvious changes to the EEG during the SOP are a progressive decrease in alpha (8–12 Hz) power, as well as a progressive increase in slow (<1 Hz), delta (.5–5 Hz), and theta (5–8 Hz) power [1], [23]–[26]. Recent intracranial recording studies suggest that this progression of EEG activity relates to changes in thalamic activity that occur prior to changes in cortical activity, the timing of which has high variability between subjects [27].

Ideally, any descriptor of sleep must account for the fact that it is a complex neural process consisting of multiple local [28]–[30] and spatiotemporally-evolving [23], [31]–[35] factors. In practice, neural activity is characterized through through polysomnography (PSG)—the visual analysis of brain (EEG), muscle (EMG), muscle (ECG), cardiac (EOG), and respiration (PTAF/Airflow) data. In current clinical practice, sleep EEG is visually scored using the Rechtschaffen and Kales (R&K) system [24], comprised of Wake, Stage 1–3 NREM sleep, and REM, defined in 30-second epochs. Researchers such as Chiappa [36] and Hori [37] found that the R&K system was too coarse to properly track the SOP dynamics, and consequently developed alternative scoring systems with many more stages, which were scored in much smaller epochs. Unfortunately, neither of these higher resolution frameworks enjoyed wide implementation, perhaps due to labor-intensive scoring rules. Additionally, existing scoring systems do not explicitly account for heterogeneity observed in normal patients as well as variability associated with age, medications, or neurological disorders [38]–[40].

Overall, current practice views the SOP in a binary semantic framework, and analyzes behavioral and physiological data independently. In this paper, we place the SOP within a physiologically and statistically principled model framework, which allows us to explicitly characterize the dynamic interaction of multiple physiological and behavioral experimental observations. Specifically, given the behavioral task and our experimental setup, we simultaneously acquired three modalities of observations related to sleep initiation: behavioral responses, EMG activity, and EEG spectral power. These observation types each contribute information across multiple time scales about different components of a subject's neural state. By combining the information from of all of these different types of observations, we can create a more robust and principled estimate of wakefulness during the process of sleep initiation that takes advantage of both behavioral and physiological data.

## Results

### A Novel Breathing-Based Behavioral Task to Track the Sleep Onset Process

In order to track the course of sleep initiation, our goal is to create a continuous-valued metric of wakefulness that is based on simultaneously observed data from multiple modalities, and for which statistical confidence can be computed. To do so, we must create a task that consists of multiple objective behavioral observations related to wakefulness, which can be tracked across the sleep initiation process. Standard behavioral response tasks that have been used previously, involving external audio, visual, or tactile stimuli, are potentially arousing and may perturb sleep initiation [17]–[19], [41]. We therefore require a paradigm free of arousing external stimuli, yet with repeated trials that can persist throughout the sleep initiation process. To solve this problem, we designed a self-regulated behavioral task centering on breathing.

Subjects were given a small, 2oz, gel-filled stress ball to hold in their dominant hand. They were instructed to breathe normally with eyes closed, and to gently squeeze the ball on each inhale and release on each exhale. Thus, each breath acts as a stimulus, and each corresponding squeeze (or lack thereof) is the corresponding response. A correct response is defined as squeeze centered on a respiratory inhale, and an incorrect response is either a lack of response or an incorrectly timed squeeze. Subjects were instructed to start the task as soon as the lights were turned out.

**Figure 1:**
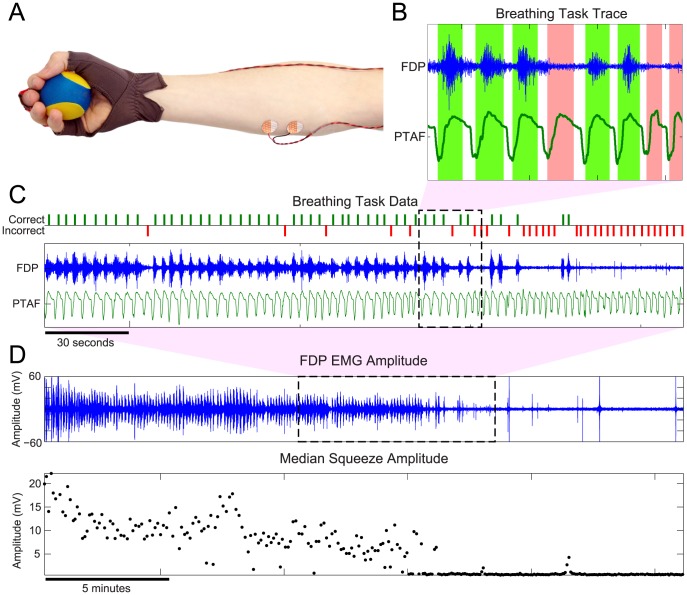
Tracking the sleep onset process with a behavioral task mediated by breathing. (A) In addition to a high-density EEG cap and standard PSG sensor array, subjects were fitted with a force-sensing glove, bipolar EMG electrodes on their flexor digitorum profundus (FDP), and a small gel-filled stress ball. Subjects were instructed to breathe normally with eyes closed as they fell asleep, and to gently squeeze the stress ball on each inhale, releasing it on each exhale. (B) Times at which a squeeze was aligned with a corresponding inhale were scored as correct trials (green regions). Times with an inhale and no squeeze (or a misaligned squeeze) were scored as incorrect trials. (C) By scoring this task across the sleep initiation period, we can track behavioral dynamics during the transition from wake to sleep, without using any external task stimuli that could produce arousal. (D) EMG amplitude during squeezes decays during the SOP. Over the course of the experiment, the FDP traces (top panel) were processed to extract the median amplitudes of each squeeze (bottom panel), which can be used as a correlate of wakefulness during the SOP.

An additional bipolar adhesive EMG sensor recorded activity of the flexor digitorum profundus (FDP) responsible for finger flexion. Subjects also were fit with a glove designed with a force sensitive resistor (FSR) embedded in the middle finger, to measure finger flexion during the behavioral task (Fig. **1**a). Both the glove and FDP EMG sensors detect even gentle squeezes (on the order of the force required for a mouse click), thereby allowing subjects to perform the task with minimal effort or muscle fatigue.

The traces from the glove and FDP EMG were time-aligned with simultaneously recorded PSG respiratory metrics (PTAF, airflow, and abdominal belt) (Fig. **1**b,c). These recordings were then visually scored in the following manner: The apex of each respiratory inhale was considered a trial. If a squeeze (visually scored using the EMG/glove activity) was present during an inhale (visually scored using the PTAF, airflow, and abdominal belt), the trial was scored as *correct* (Fig. **1**b, green regions). If there was no visible response or a misaligned squeeze, the trial was scored as *incorrect* (Fig. **1**b, red regions). Periods including motion artifacts, signal degradation due to temporary sensor disconnection, or any other uncertainties in the signal were left unscored and treated as missing data in subsequent analyses. Scoring was started at the first sequence of trials following lights out that began with at least 3 consecutive correct responses. Scoring was stopped 10 minutes following the last correct response. Some subjects reported difficulty performing the task while they adjusted to wearing the full EEG/EMG/PSG montage. After excluding data from four nights with poor task compliance due to difficulty habituating to the extensive sensor montage, the remaining 16 nights from 9 subjects were processed using our algorithm. A wake probability curve was generated for each night.

### Simultaneous Observations Provide a Robust Framework for Tracking the Sleep Onset Process

Along with each behavioral response, we simultaneously observed the EMG activity in the FDP muscle—including the amplitude of each squeeze accompanying a correct response (Fig. **1**d). To measure the magnitude of the squeeze, we computed the amplitude envelope of the EMG using a Hilbert transform, then calculated the mean amplitude in a 1 second window centered around the trial time. In tracking EMG data over the course of the SOP, we see that, like a continuous measurement of the muscle activity during a dead man's switch paradigm [1] the EMG squeeze amplitudes decay until the correct responses stop entirely (Fig. **1**d, bottom panel). Thus, the EMG squeeze amplitudes provide a continuous-valued metric of both muscle tone and of wakefulness.

Paired with the behavioral task, we simultaneously recorded EEG data from each of the subjects. For our analysis, we chose to focus on the most straightforward, continuous correlates of sleep in the EEG: the power in delta, theta, and alpha bands. The power in these bands contributes information about different neurophysiological systems in play during the SOP.

With all these sources of information, we can devise a method for integrating them into a single, statistically principled model of wakefulness during the SOP.

### An Empirical Wake Probability Model of Sleep Onset Process Dynamics

Our modeling approach centers on the idea that the EMG, EEG, and behavioral observations each provide information related to the activity of different physiological systems involved in different aspects of the SOP. By integrating the information across these systems, we can create a robust framework for tracking the dynamic changes in a subject's wakefulness as they fall asleep. In this section, we provide a non-technical summary of our modeling methodology and its rationale. We describe the mathematical formulation of our approach in detail in the *Materials and Methods* section, *Formulation of the Wake Probability Model of the Sleep Onset Process*.

We model sleep onset dynamics relative to the observed behavioral, EEG, and EMG data. Our *wake probability model* states that as the SOP progresses from wake to sleep:

•Probability of a correct behavioral response decreases

•EMG squeeze amplitude decreases

•Alpha power decreases

•Theta power increases

•Delta power increases

**Figure 2:**
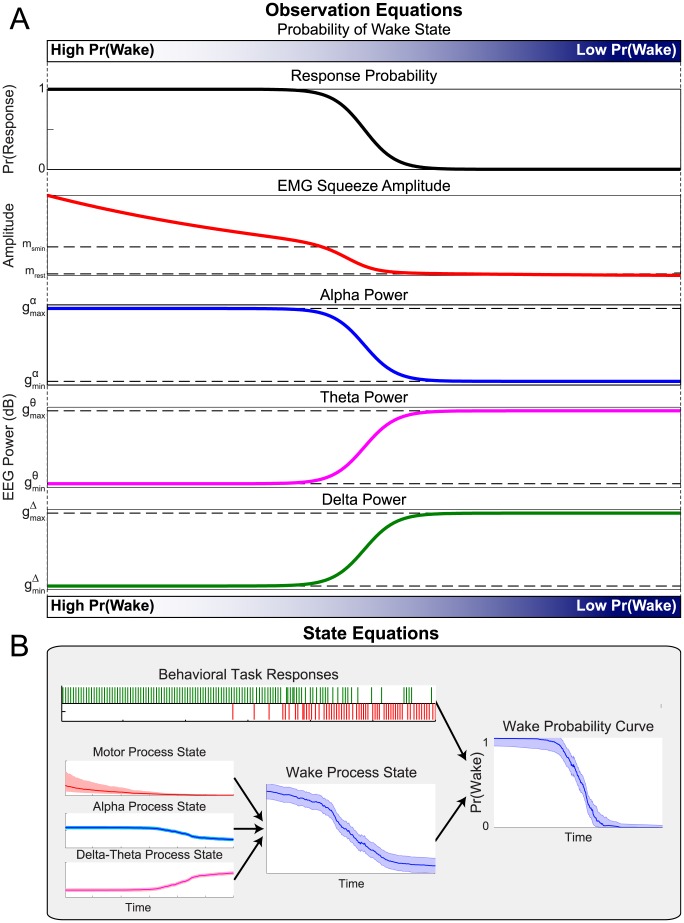
A data-driven model of sleep onset process dynamics. (A) As the SOP progresses, Pr(Wake), the probability that the subject is awake, decreases. As Pr(Wake) decreases, the probability of a correct behavioral response, the EMG squeeze amplitude, and the alpha power will also decrease, while the delta and theta power will increase. (B) We can then use experimental data to estimate Pr(Wake) over time. We define state processes representing the activity of the systems underlying the motor, alpha power, and delta-theta power observations. The combined information from all states represents the level of all activity related to waking, and is used along with the behavioral task responses to estimate the wake probability curve, which tracks Pr(Wake) over time.

A schematic of this model is shown in Figure **2**A.

In our model, we define the *wake probability* Pr(Wake) as the distribution of the posterior probability (the probability of the model given the observed data) that the conditions necessary for the wake state are met: the subject is responding correctly, the EMG amplitude and alpha power are at their highest, and delta power and theta power are at their lowest. Therefore, as these conditions are met, the mode of Pr(Wake) approaches 1. This allows us to use Pr(Wake) as a metric representing the degree to which we believe the subject is awake. Moreover, we have formulated Pr(Wake) so that it also represents the distribution of the instantaneous probability a correct behavioral response, and thus directly interpretable in terms of standard behavioral paradigms. The wake probability model can be fit to experimental EEG, EMG, and behavioral data to track Pr(Wake)over time. We call the time-varying estimate of Pr(Wake) the *wake probability curve*. We describe wake probability in the *Materials and Methods* section, *Interpreting Wake Probability*.

To implement this approach, we use a Bayesian state-space modeling framework [6], [42]–[44] (Fig. **2**B). State-space modeling allows us to estimate something that is not directly observable (in this case, the probability of the subject being awake) from observations that can be directly measured (in this case, the EEG, EMG, and behavioral data). We first model the observations as a function of state processes that represent, in abstract, the level of activity in each of these systems (see *Materials and Methods*, *State Models*). These state processes are not directly observable, but their values can be inferred from the data given the structure of the model. We create three state processes: a motor activity process state *x^m^*, an alpha process state *x^α^*, and a delta-theta process state *x* ^Δ*θ*^. For each of the state processes, we define a *state equation*, which describes the way the states evolve over time. The state equations are designed to reflect the notion that states cannot change instantaneously, and that they are related to their past values.

The motor activity process *x^m^* represents the degree of wakefulness estimated from the amplitude of the EMG during the behavioral task (Fig. **1**d). As the subject becomes drowsy, the force of the squeezes will decrease and eventually revert to the underlying muscle tone.

The alpha process *x^α^* represents the degree of wakefulness estimated from the spectral power in the EEG alpha band. As the subject falls asleep, the alpha power will decrease. If the subject awakens, the alpha will return (subjects are told to maintain eyes closed).

In our model, the delta-theta process *x* ^Δ*θ*^ represents the degree of wakefulness estimated from the spectral power in the EEG delta and theta bands. As the subject enters NREM sleep, the delta and theta will increase. If the subject awakens, the power in delta and theta will rapidly decrease.

Each of the state processes can change independently, reflecting the asynchronous dynamics of the cortical and subcortical systems generating these EEG rhythms throughout the SOP. We formulate our model of wake probability to be a function of the linear combination of the three states such that *x^m^* and *x^α^* have a direct relationship to Pr(Wake), while *x* ^Δ*θ*^ will have an inverse relationship to Pr(Wake).

We next define the *observation equations* (Fig. **2**A), which describe mathematically the relationship between the experimental observations (EMG, alpha, delta, theta, and binary responses) and the underlying state processes (see *Materials and Methods*, *Observation Models*). Each observation equation is constructed so that the value of the state process is high when the data indicates high activity, and low when the data indicates low activity. We also define an observation equation relating behavioral response to wakefulness, such that response probability is directly proportional to Pr(Wake).

Together, the state and observations define a framework relating our experimental observations to the underlying behavioral and physiological processes, and provide an explicit model for how the aggregate activity of these processes relates to changes in behavior.

Using the state and observation equations together with the data, we simultaneously estimate the hidden states and model parameters at each time, using a particle filter, which is a Bayesian sequential importance resampling method (see *Supplementary Materials, Particle Filter*). The particle filter evaluates all the data observations in context with model equations and computes the maximum-likelihood state and parameter values. The particle filter output is an estimate the full distribution of the posterior probability of the wake probability model, given the observed EEG, EMG, and behavioral data.

In summary, our approach takes basic assumptions about the way experimental data evolves during the SOP and explicitly models them in a state-space framework. From this model, we can estimate the wake probability curve, which tracks the dynamics of the SOP by integrating simultaneously observed behavioral and physiological data. Thus, our method provides a robust, statistically-principled, and physiologically-motivated method for characterizing SOP.

### Comparing SOP Dynamics for Subjects and Populations

Since subjects fall asleep at different rates with different dynamics, comparing physiological activity between subjects and populations has been a difficult problem. As a result, previous studies have been limited to anecdotal analyses or static statistical analysis using categorical bins for data. Fortunately, the wake probability now allows us to compare the SOP of different subjects in a principled manner. This is because the value of Pr(Wake) provides a common point of wakefulness for the alignment of the physiological data across subjects. To characterize the population dynamics of the EEG during the SOP, we estimated the EEG spectrum of the population as a function of Pr(Wake). Specifically we calculated the median spectrum over all subjects and nights at each value of Pr(Wake). We considered values of Pr(Wake) in discrete bins of width 0.0025 between 0 and 1. We then plotted this group-level spectrum as a function of Pr(Wake). The result is a visualization that looks like a spectrogram, but displays median population spectral power as a function of frequency and Pr(Wake), rather than frequency and time. We refer to this plot as the *SOP population spectrogram*. Since Pr(Wake) also represents response probability, this analysis therefore characterizes the average EEG spectrum dynamics during the SOP as the behavioral response probability declines during the transition from wakefulness to sleep.

The SOP population spectrogram allows us to summarize an SOP phenotype for a given population of subjects. Furthermore, we can characterize the difference in the SOP phenotype of two populations by comparing their population spectrograms. To do so, we performed a bootstrap procedure [45], [46] to compute the difference distribution for each frequency-Pr(Wake) bin using 10,000 iterations per bin. A *frequence* × Pr(Wake) bin was said to be significantly different between populations if zero fell outside the 2.5^th^ and 97.5^th^ percentiles of the difference distribution.

The procedure for constructing an SOP population spectrogram is described in detail in the *Materials and Methods* section, *Computing SOP Population Spectrograms*.

### Goodness-of-Fit

Since wake probability is a useful abstract quantity not directly observable during the SOP, standard analyses of measurement error are not possible, as there is no ground truth against which Pr(Wake)can be compared. Instead, we can perform a likelihood analysis to assess how well a particular model of the SOP of describes the behavioral task data. We used Bayesian Monte Carlo procedures to compute the likelihood of a given model as well as compare the likelihoods of two competing models. These procedures are described in detail in the *Supplementary Materials* section, *Bayesian Goodness-of-Fit*.

Clinically, the SOP is typically characterized by hypnogram-based definitions of a single moment of sleep onset. By definition, any characterization of a “sleep onset point” cleaves SOP dynamics into a unitary wake state prior to the sleep onset point and a unitary sleep following that point. Thus, while never stated outright, any definition of a sleep onset point imposes an instantaneous transition model on the SOP. Since these models assume an instantaneous wake/sleep transition, it follows that they also assume an instantaneous change in behavioral task performance. We can therefore construct a probability curve analogous to the wake probability curve for any instantaneous transition model by conservatively assuming that the subject should respond correctly with significance (95% accuracy) when deemed awake, and incorrectly with significance (5% accuracy) when deemed asleep. We can then compare these curves to the wake probability curve in order to assess the relative goodness-of-fit. The specifics of the Bayesian construction are also detailed in the *Models* section.

To perform the goodness-of-fit analysis, we computed the likelihood distributions for the wake probability model and four different instantaneous transition models using the behavioral data across all subjects for all nights. We then computed the confidence (Bayesian credible interval) with which the wake probability model likelihood differed from that of each instantaneous transition model. Since the wake probability model incorporates information from the behavioral data, we used the posterior distribution from the time step prior to the behavioral observation in all of the goodness-of-fit analyses to insure that use of true behavioral response in the wake probability model formulation did not unfairly affect the results.

### Modeling the Dynamics of Behavioral and Physiological Observations during Sleep Onset Process

**Figure 3:**
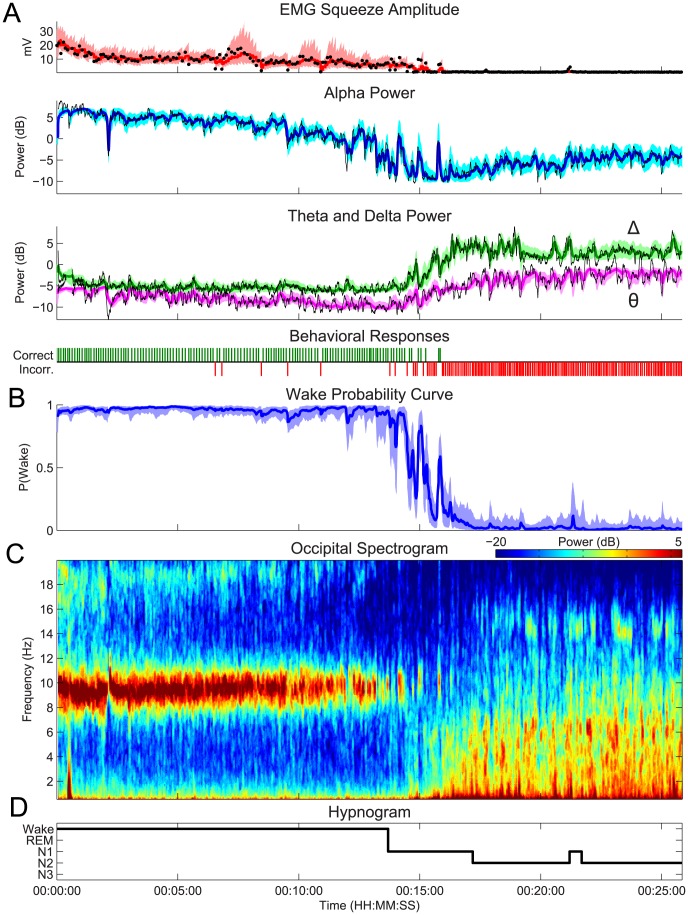
Fitting the model to experimental data during the course of sleep initiation. (A) The simultaneously observed EMG (black dots), and EEG observations (black curves), and behavioral responses (green  =  correct, red  =  incorrect) from the experiment are used to estimate the wake probability curve (B), which shows Pr(Wake), the probability of a correct response given the EEG and EMG data, over time. The wake probability curve acts as a statistically principled means of tracking the dynamics sleep initiation, and agrees well with features of the EEG spectrogram (C), the clinical hypnogram (D). In (A) the model estimates of the EMG and EEG mean (curves) and 95% confidence intervals (shaded regions) are shown (EMG: red, alpha: cyan, theta: magenta, delta: green). For all state estimates, we compute the distribution medians (colored curves), and 95% confidence intervals (shaded regions) of the model estimates.

In our model of the SOP, a subject's probability of wakefulness is based the combined information from both behavioral and physiological observations. Figure **3** shows an example of the model fit to data from one of our experimental subjects. The wake probability curve (Fig. **3**B) is estimated using information from both behavioral and physiological data (Fig. **3**A, EMG: black dots, alpha, delta, theta power: black curves), and therefore integrates features of both modalities. This is most clearly demonstrated by comparing the wake probability curve with the corresponding raw data (Fig. **3**A), EEG spectrogram (Fig. **3**C), and clinical hypnogram (Fig. **3**D).

The behavioral data starts with a train of correct responses while the subject is awake with eyes closed. This period is followed by increasing numbers of incorrect responses, which coalesce into a train of incorrect responses. Correspondingly, the EEG data transitions from a pattern with a strong alpha oscillation and minimal delta or theta during high wakefulness, to a pattern with intermittent alpha and rising energy in delta and theta bands. Eventually, the EEG is dominated by delta and theta power, and the alpha oscillation disappears. During the SOP, we observe the alpha power decreasing in a sigmoidal fashion, and the delta and theta power increasing in a sigmoidal fashion. The EMG amplitude decays exponentially at first, followed by a sigmoidal trajectory. These trajectories are in line with our model observation equations, depicted in (Fig. **2**A). Consequently, the model estimates (Fig. **3**A, colored curves and regions) track the raw data (black) closely.

The structure of the wake probability curve (Fig. **3**B) appears to successfully integrate features of the behavioral and physiological observations. For roughly the first 13 minutes of the SOP, the wake probability curve is close to 1, with a narrow confidence interval. This agrees with large number of correct behavioral responses, strong alpha mode in the spectrogram, and hypnogram score of wake. Shortly after 13 minutes into the SOP, the probability curve fluctuates several times before settling into a low Pr(Wake) median at around 17 minutes. After 17 minutes, the behavioral responses are exclusively incorrect, the EEG alpha power has dropped out, there is a sharp rise in delta and theta power, and Pr(Wake) is low. Moreover, the rise in Pr(Wake) between 21 and 22 minutes aligns directly with the hypnogram, which goes from Stage 2 to Stage 1 and back during the same interval.

### The Wake Probability Model Tracks the Dynamic Transition between Wake and Sleep

**Figure 4:**
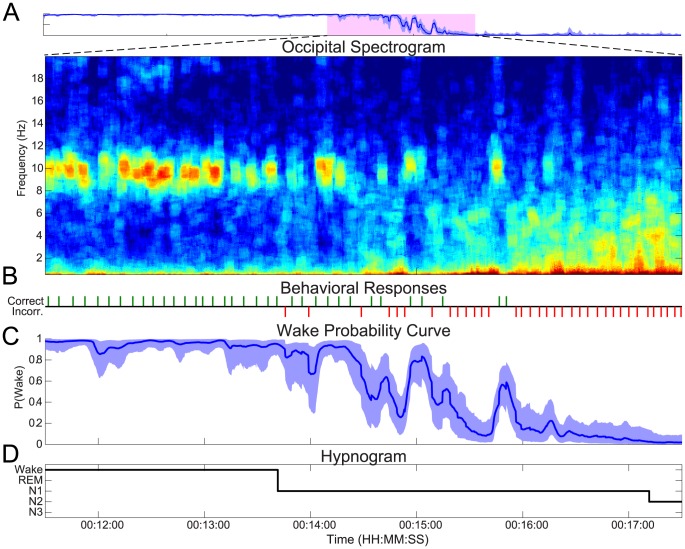
A close up of the sleep/wake transition period of the SOP from the same subject from Figure 3 illustrates the interplay of the EEG and behavior during the SOP. The corresponding (A) spectrogram, (B) behavioral responses, (C) the wake probability curve, (D) and the clinical hypnogram are shown. In (C), the distribution median (curve), and 95% confidence intervals (shaded region) are shown.

By examining the transition period in this same subject in greater detail, we can gain further insights into how the behavioral and EEG data are combined to estimate the wake probability curve. Figure **4** shows a close up of the data from Figure **3** on a time scale of 6 minutes. This period begins with a string of 22 consecutive correct behavioral responses (Fig. **4**B). Since correct responses indicate wakefulness, this information pushes Pr(Wake) towards 1. During the same time, however, the alpha power (Fig. **4**A) is sporadically present, supplying support that the subject is more ambiguously awake (i.e., less than 1). Given our model, low alpha and high delta-theta power pull Pr(Wake) towards 0. While the behavioral responses are correct, loss of alpha power indicates reduced wakefulness, resulting in a lowering of Pr(Wake) and an increase in the uncertainty of the estimate (as indicated by wider confidence bounds).

As the subject transitions through the SOP, the number of incorrect responses increases, the alpha diminishes progressively, and delta and theta appear and begin to coalesce into prominent oscillations. This period is marked by an alternation between alpha and delta/theta activity [12], [26], [32], and continues until the alpha is gone, the delta/theta is high, and all the responses are incorrect. Consequently, we see peaks in Pr(Wake) where there is high alpha power, low delta/theta power, and correct responses, and troughs in Pr(Wake) where the opposite is true. The magnitude of these peaks and troughs are based on the degree to which the aggregate data indicates that the subject is awake. The confidence bounds are related to the degree to which all of the data is in agreement. In comparison to the clinical hypnogram (scored in 30 s epochs) (Fig. **4**D), the wake probability curve characterizes this transitional period of the SOP with a much higher temporal resolution. Additionally, the wake probability curve describes a continuum of wakefulness, whereas the hypnogram discretizes this transitional period into three categories: Wake, Stage N1, and Stage N2 states.

**Figure 5:**
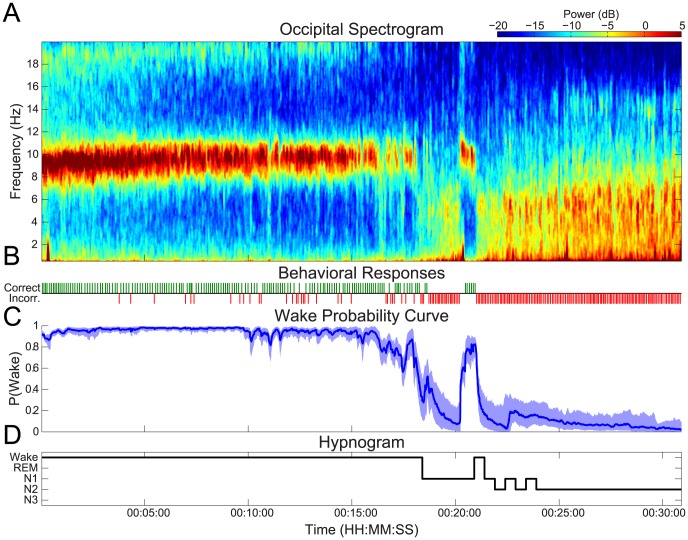
Tracking a fragmented SOP. A comparison of the (A) spectrogram, (B) behavioral responses, (C) the wake probability curve, (D) and the clinical hypnogram. In (C), the distribution median (curve), and 95% confidence intervals (shaded region) are shown. The probability of wakefulness tracks both the gradual time course of the initial descent into sleep, as well as the rapid changes during the arousal period.

The transition from wake to sleep can be fragmented—most notably in patients suffering from difficulties with sleep initiation, but also in healthy people. Figure **5** shows data from the second experimental night from the same subject shown in Figures **3** and **4**. Rather than the smooth transition seen the first experimental night, we observe that this night the subject had brief arousal period in the middle of the SOP. The wake probability curve captures both the slow transition from wake to sleep, as well as the rapid changes in wakefulness during the arousal period.

As in the subject's first night, the SOP begins with trains of correct responses (Fig. **5**B), a strong alpha mode, and low delta and theta (Fig. **5**A), which results a high Pr(Wake) (Fig. **5**C). The alpha mode then becomes sporadic, which coincides with an increase in incorrect responses. Next, there is a train of consecutive incorrect responses, the alpha mode disappears, and there is a dramatic increase in the theta power and rising delta power. Consequently, the median of Pr(Wake) drops towards 0.

Suddenly the correct responses begin again, the alpha mode returns, and the delta and theta drop off. Given this information, Pr(Wake) then ascends rapidly towards 1. After approximately 1 minute, the responses become exclusively incorrect, the alpha power disappears. The delta and theta power rapidly return to their pre-arousal levels, continuing to increase for the rest of the SOP. The wake probability curve tracks the drop in Pr(Wake) and the dynamics for the rest of the SOP. Again, the wake probability curve structure agrees strongly with the structure of the hypnogram (Fig. **5**D), but provides greater temporal resolution and finer granularity in the state estimate.

### Characterizing Heterogeneity of Sleep Onset Phenotypes: Alpha Dropout Prior to Cessation of Behavioral Response

In the preceding illustrative examples, there is strong agreement between the behavioral and physiological data. In practice, however, there is neurophysiological heterogeneity observed—even within healthy subjects—such that there is often a great disparity between behavioral and physiological metrics of sleep onset. In this section, we show how the wake probability curve characterizes such situations.

**Figure 6:**
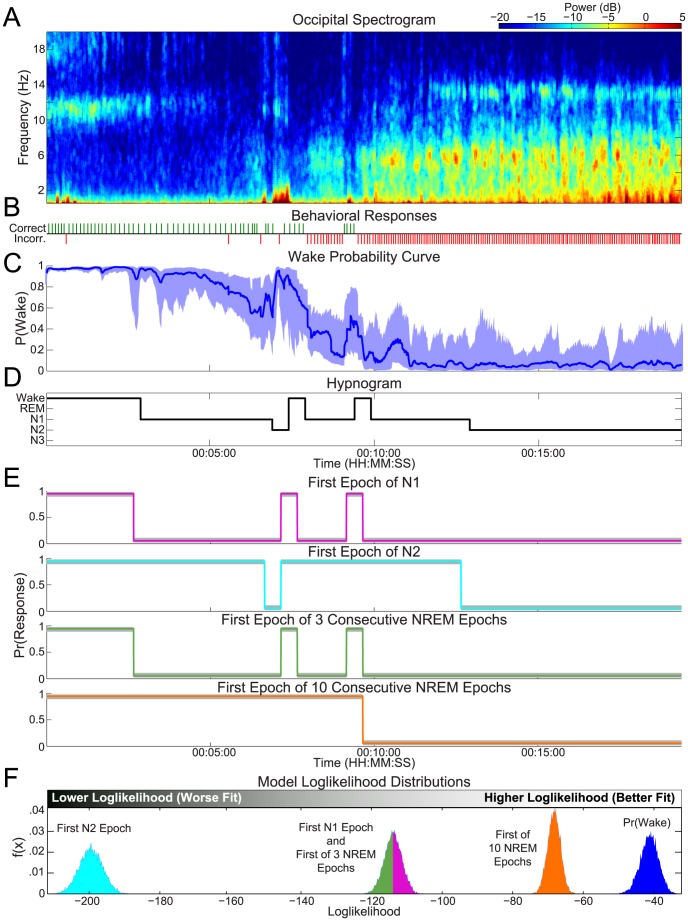
Heterogeneity in healthy subjects: An SOP phenotype with alpha power dropout before the cessation of behavioral activity. The (A) spectrogram, (B) behavioral responses, (C) the wake probability curve, (D) and the clinical hypnogram are shown for a subject with this SOP. The wake probability curve captures persistence of behavioral responses after alpha power declines, a feature that is not evident in hypnogram-based binary models of sleep onset (E). The Bayesian likelihood analysis (F) shows that wake probability significantly outperforms (99.99% Bayesian credible interval of the difference distribution falls above zero) all of the instantaneous transition models in the ability to correctly predict the behavioral responses for this subject.

Figure **6** shows the results from another healthy subject with a dramatically different SOP phenotype. As in the previous case, the experiment begins with a strong alpha oscillation, which eventually disappears (Fig. **6**A). In this case, however, the correct responses persist long after the alpha has diminished (Fig. **6**A). Moreover, there is a roughly 5-minute interval between the time the alpha mode declines and the time the theta and delta power increase.

This SOP *alpha dropout* phenotype with a long interval between alpha power decline and delta/theta power rise results in disagreement between standard sleep scoring and a behavioral analysis. In this period between the loss of alpha and loss of response, the hypnogram (Fig. **6**D) describes the subject as being predominantly in Stage N1, with a brief period of Stage N2, and a short period of Wake when there is a short increase in alpha. Thus a standard interpretation of the hypnogram would place sleep onset at the first epoch of Stage N1, approximately 3 minutes into the SOP. This is in contrast to the behavioral data, which continues to indicate wakefulness for another 5 minutes past the first epoch of Stage N1. The wake probability curve (Fig. **6**C), however, integrates all the data such that the estimated median of Pr(Wake) is still high during this period, declines slightly, and has a large uncertainty as a result of the contradicting observations.

By combining both the behavioral and physiological data into the estimate of Pr(Wake), we can bridge the disparity seen between metrics that exclusively rely on ether behavior or EEG alone. The result is a model that can represent deviation from the population norm as increased uncertainty.

In this analysis, 2 of the 9 subjects (*Supporting Information* Figures S1 and S2) clearly exhibited this *alpha dropout phenotype*, in which alpha power declined up to several minutes prior to the termination of correct responses and the increase of delta and theta power. For both subjects, this phenotype was present on both experimental nights. Three of the four nights had periods of scored Stage N1 during which there were correct behavioral responses. In none of the cases did we observe correct responses in the presence of strong delta and theta. This suggests that loss of alpha power, while necessary, is not sufficient for the loss of behavioral responses.

### Wake Probability Outperforms Clinical Models of Sleep Onset

In clinical practice, the most common definitions for the moment of sleep onset are: the first epoch of Stage N1, the first epoch of Stage N2, the first of any 3 consecutive NREM (N1 or deeper) epochs, and the first of any 10 consecutive epochs of NREM. Though not stated explicitly, any characterization of a point of sleep onset actually imposes a model on the SOP with an instantaneous sleep/wake transition, which does not agree with the continuous, dynamic transitions observed in the data. We performed a likelihood analysis comparing how well of the wake probability model and instantaneous transition models fit the behavioral data. Likelihood is a relative estimate of goodness-of-fit, and given two competing models, the one with the better fit will have a higher likelihood.

**Figure 7:**
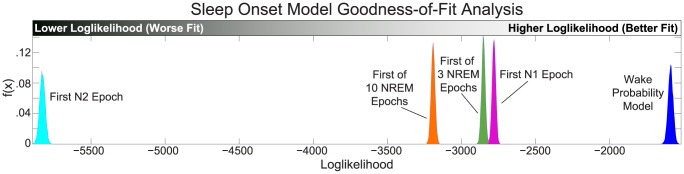
Goodness-of-fit analysis of the wake probability model versus instantaneous transition models. (A) Using a Bayesian Monte Carlo analysis, we compute the distribution of the total loglikelihood of each of the models given the behavioral task data across all subjects and all nights. The wake probability model significantly outperformed all of the instantaneous transition models (99.99% Bayesian credible interval of the difference distribution fell above for all models).

The comparative likelihood analysis showed that the wake probability model significantly outperformed each of the instantaneous transition models with at least 99.99% confidence. These results are summarized in Figure **7** and in Table 1. Overall, the wake probability model fit the data the best with the largest median loglikelihood (−1589), followed by, in order of goodness-of-fit, the first epoch of N1 model (−2781), the first of 3 NREM model (−2852), the first of 10 NREM model (−3191), and by the first epoch of N2 model (−5828).

**Table 1:** Bayesian goodness-of-fit analysis results.

Model	Goodness-of-fit Rank	Loglikelihood Median	Loglikelihood 95% Credible Interval	Difference Distribution Median	Difference 95% Credible Interval	Credible Interval for Difference>0
**Wake Probability Model**	1	−1589	[−1623 −1556]			
First N1 Epoch	2	−2781	[−2805 −2757]	1191	[1149 1234]	99.99%
First of 3 NREM Epochs	3	−2852	[−2877 −2828]	1263	[1211 1306]	99.99%
First of 10 NREM Epochs	4	−3191	[−3217 −3165]	1630	[1559 1645]	99.99%
First N2 Epoch	5	−5828	[−5864 −5792]	4239	[4189 4288]	99.99%

Summary of Bayesian goodness-of-fit analysis using the data from all subjects and all nights, comparing the wake probability model vs. several common instantaneous transition models of sleep onset. The analysis results are ranked in order of how well they fit the behavioral data (the greater the log-likelihood, the better the fit). The loglikelihood distribution characterizes how well each model is able to the behavioral data. The difference distribution (wake probability loglikelihood – instantaneous transition loglikelihood) describes the performance improvement of the wake probability model over the competing model. The credible interval with which the difference distribution is above zero reflects the confidence in which we believe the wake probability model outperforms the competing model.

To illustrate the way in which the wake probability model improves upon the instantaneous transition models, we performed the goodness-of-fit analysis on a single night of data. Figure **6**E and F show, respectively, the instantaneous transition model response probabilities generated from the hypnogram, and the resultant goodness-of-fit analysis for that experimental session. This clearly shows the way in which the instantaneous transition models implicitly discretize complex dynamics of the SOP into unitary “wake” and “sleep” states, thus losing the ability to capture any nuances in state throughout. Furthermore, since current EEG-based definitions of sleep onset do not include behavioral information, the assumption that Stage N1 is equitable with “sleep” can be misleading [1], particularly for those subjects (like this one) in which behavior persists past the alpha dropout.

Consequently, the wake probability model (C) fit the behavioral response data the best (F) with median loglikelihood of −41—significantly outperforming the instantaneous transition models with at least 99.99% confidence. Within the class of the instantaneous transition models (E), the first of any 10 consecutive NREM epochs model performed the best in this particular case, with a median loglikelihood of −68. In this case, the first epoch of N1 model and first of 3 consecutive NREM epochs model both provided the same response probability estimates, and each had a median loglikelihood of −113. Finally, the first epoch of N2 model performed the worst, with a median loglikelihood of −199.

Overall, these results suggest that the wake probability model is a more mathematically and physiologically appropriate metric with which to track the SOP than are the current hypnogram-based metrics.

### Population Analyses Quantify SOP Phenotype Differences

One of the key strengths of the wake probability model is that it can characterize the EEG activity for groups of subjects across the entire SOP, rather than at a single point of alignment. Using the wake probability curves from multiple subjects, we can compute an *SOP population spectrogram*, which plots the median cross-subject EEG power spectrum as a function of the behavioral response probability (see Materials and Methods: Computing SOP Population Spectrograms). By using these techniques, we can group the SOP data of multiple subjects on a continuum, from which we make rigorous statistical statements about the differences between populations. As an example, we quantify, for the first time, the differences in the EEG between subjects with “normal” and “alpha dropout” SOP phenotypes on a continuum of wake/response probability.

**Figure 8:**
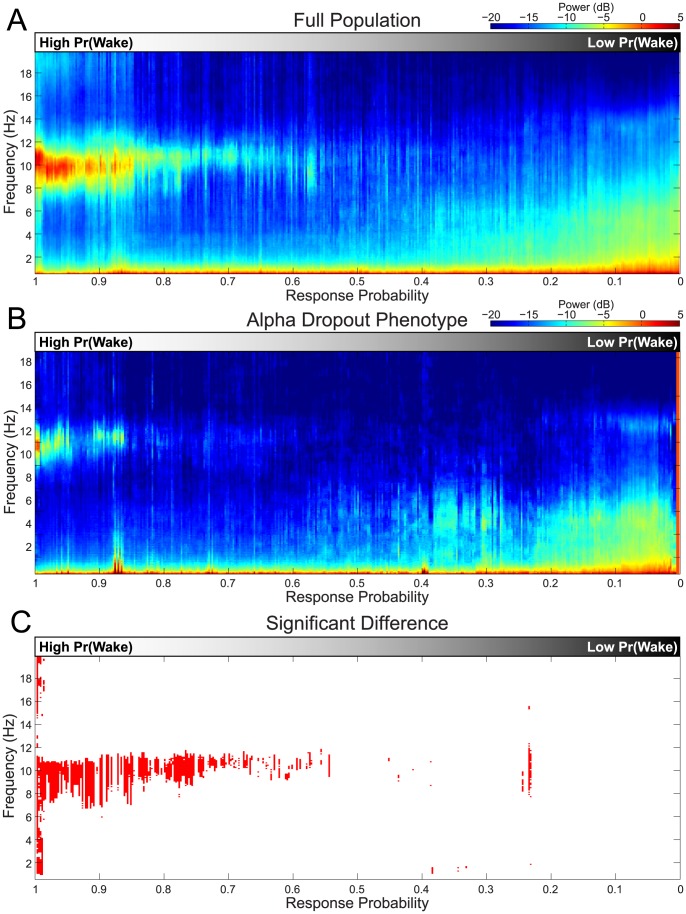
Comparing SOP phenotypes. The SOP population spectrogram visualizes EEG power spectrum as a function behavioral response probability/wake probability. (A) Using the wake probability curves to align across subjects, the median EEG spectrum as a function of behavioral response probability for the full population of subjects. (B) The population analysis is also performed for the two subjects (4 nights) showing the alpha dropout phenotype. (C) A bootstrap analysis is performed to compute the time-frequency regions where response probabilities at which the two groups differ in their spectral power.

We computed the SOP population spectrogram using the data from all the subjects and nights (Fig. **8**A). These results clearly show the dynamic transition from a strong alpha mode to increasing delta/theta power as the probability of response progresses from 1 to 0 as the subject falls asleep. As the SOP progresses, the alpha power reduces amplitude, dropping out near a response probability of 0.55. The delta/theta mode emerges at around a response probability of 0.4, increasing its bandwidth and amplitude as the response probability approaches 0.

We can also use the SOP population spectrogram to characterize difference the average EEG activity from different populations at moments at which their behavior is identical. As an illustrative example, we computed an SOP population spectrogram using the data from the two subjects (2 nights/subject, 4 nights total) that showed a clear alpha dropout phenotype (Fig. **8**B). The analysis reveals a different spectral structure, with the alpha mode dropping out near a response probability of 0.85, and delta/theta power emerging around a response probability of 0.2.

We then used a bootstrap procedure to compare the SOP population spectrograms of the subsets of subjects with normal and alpha dropout phenotypes (Fig. **8**C). This analysis revealed a region of 95% significant difference (red areas) covering the bounds of the alpha mode of the standard phenotype. These results suggest that are indeed subjects that possess significantly reduced alpha power yet can maintain behavior response levels identical to other subjects with a strong alpha oscillation. Analyses such as these therefore provide a principled mathematical framework for characterizing individual SOP phenotypes, as well as for quantifying SOP heterogeneity.

## Discussion

### The Breathing Task Could Facilitate Behavioral Tracking during the Wake/Sleep Transition

There is currently is no behavioral monitoring standard in sleep medicine.

In experimental sleep studies, active behavioral monitoring requires potentially arousing auditory stimuli. Our new breathing task presents a new paradigm for behavioral monitoring free of external stimuli. Moreover, with our new paradigm, all that is required is a respiration monitor and EMG leads on the subject's forearm, both of which are already part of the standard clinical setup. There is also no need to tackle the difficult problem of determining the correct stimulus volume that best balances salience with the potential for subject arousal.

Further experimentation is needed to definitively ascertain the comparative benefits of the breathing task over standard active behavioral measures. However, there is significant evidence in the literature suggesting that this paradigm has major advantages. While the breathing task is like all other active tasks in the sense that it requires repeated behavioral responses to stimuli, it is innovative in that there are no external sensory stimuli, which can cause arousal from sleep [18], [19], [41], [47]. Rather, this task could be said to rely on “internal stimuli” generated from the act of breathing. The breathing task therefore acts as a bridge between active and passive behavioral measures of sleep onset—providing high temporal resolution while minimizing the effects from the stimuli.

It is then a question as to whether the act of concentrating on breathing is arousing in and of itself. On the contrary, focused repeated breathing has been shown to reduce anxiety and tension [48], to decrease heart rate and blood pressure [49], to increase parasympathetic and decrease sympathetic activation [50], to decrease oxygen consumption [51], and has been correlated with reduction in EEG alpha power [52]. Since many of these effects are physiological hallmarks of the SOP, the act of attending to the breathing task would be unlikely to arouse subjects by itself, and could even potentially facilitate the wake/sleep transition. Additionally, interventional cued breathing studies have been shown to reduce the duration apnea events [7].

### The Wake Probability Model

In our model, we compute wake probability, an estimate of the probability that the subject is awake given evidence from simultaneously observed EEG, EMG, and behavioral data. This approach improves on contemporary staging of data, where a choice needs to be made between wake and sleep. Here we produce a continuous-valued metric that tracks the full spectrum of states during the SOP. In so doing, we more accurately characterize the SOP as a dynamic system, and can therefore make more precise observations and predictions about the underlying physiology. There are several key factors in this analysis that enable this dynamic, multimodal characterization.

First, we designed the wake probability model with the goal of tracking the dynamics of a gradually changing system. In his 2001 review, Ogilvie comprehensively and persuasively enumerated the preponderance of scientific evidence supporting the notion that the SOP is a gradual dynamic process, and decried the notion of characterizing a single moment of sleep onset. In the decade following, newer studies have only added more support to this argument through the further analysis of cortical and subcortical activity [27], [30]. Moreover, our nightly experiences with falling asleep tell us that the transition from wake to sleep is not an instantaneous process. In spite of all this experimental and empirical evidence, SOP dynamics have not been embodied in previous quantitative analyses. By modeling wake probability as a continuous-valued metric, we can now characterize the SOP as a dynamic process, bridging the gap between the evidence and the analysis techniques.

Second, our model incorporates data from both physiological and behavioral observations. Often, there can disagreement between EEG and behavioral metrics of in the estimation of sleep onset, since changes in the EEG and behavior are not necessarily time-locked to each other. Ogilvie observed that behavioral responses could persist well into Stage N1—far beyond the point at which many standard criteria for sleep onset would consider sleep—and went so far as to suggest that N1 not be even considered to be true sleep [1]. Additionally, visual scoring paradigms have difficulty handling the heterogeneity observed in the normal EEG population, and consequently will deem a subject to be asleep due to diminished or missing alpha oscillations. It is therefore is of vital importance to use both behavioral and physiological data in any metric that characterizes the SOP.

Third, we model the SOP as a combination of multiple independent components, which can evolve on different time scales. In formulating the model, we designed the state equations so that the alpha, delta/theta, and motor states could evolve independently based on the data. This flexible setup reflects the idea that interacting systems can activate or deactivate on different timescales throughout the SOP, an idea substantiated through intracranial studies of corticothalamic activity [27]. In our model, is the superposition of these states that governs the behavioral response data and vice versa. In our model, each observation type reflects the activity of a systems-level neural component of the SOP, and the aggregate effect of all the systems governs arousal and consequently behavior.

Finally, our framework is statistically principled. Since the model is Bayesian and computes the full posterior distribution of Pr(Wake), we can perform many other rigorous statistical comparisons between any set of points in a night for a single subject, as well as comparisons between subjects [53], [54]. Moreover, if a single point of alignment is indeed required, we can now take a statistically principled approach by defining it using Pr(Wake). For example, one could identify the first time point at which Pr(Wake) was significantly less than 0.95.

### Wake Probability vs. Sleep Probability

In this method, we frame the characterization of the SOP in terms of the probability of wakefulness, rather than the probability of sleep. We do this because the SOP is a complex multifocal process [28], [29], [32], which is constantly evolving. Consequently, trying to estimate the probability of sleep is the equivalent of shooting at a moving target, since “sleep onset” could refer to any point on a vast continuum of dynamic neural activity. To simplify the problem, we therefore chose to create a simple model of the waking state and track its disappearance rather than tackle a complex model of sleep and track its initiation. It should be noted that Pr(Wake) does not necessarily equal 1−Pr(Sleep), as local sleep-related processes can co-exist with wake-related processes in the brain during the SOP [32]. Additionally, this framework lets us define wake probability as equivalent to probability of a correct response, so that Pr(Wake) can be discussed in terms of behavioral responsiveness, given the additional data from the EEG and EMG.

### Characterizing the SOP across Subjects and Phenotypes

#### Wake probability provides principled alignment for quantitative cross-subject comparisons

A major innovation of our method is the ability to make an apples-to-apples quantitative comparison between sets of subjects using the SOP population spectrogram. In our analyses, we observed a subset of subjects for which behavioral responses persisted for several minutes after loss of alpha power. While subjects with low or missing alpha were first noted by the Davis group [26] in the late 1930s, and Ogilvie and Wilkinson observed subjects responding to reaction-time tests during Stage N1 [12] in the 1980s, there has been, until now, no formal statistical paradigm with which the physiological and behavioral data could be aligned in a unified, continuous framework for quantitative cross-subject analysis. By using the SOP population spectrogram, we are finally able to state with statistical certainty that, for periods with the same instantaneous behavioral response probability, there exists a subset of healthy subjects with significantly lower alpha power than the normal phenotype subjects.

#### Understanding alpha dropout

A possible mechanism for the alpha dropout SOP phenotype could relate to work by Magnin et al. (2010), in which intracranial EEG measurements during the SOP revealed that thalamic circuits tend to change state several minutes before cortical circuits. The latency between changes in thalamic and cortical activity showed substantial variability between subjects. Since it is well known that alpha oscillations are generated when thalamic relay circuits are placed in a depolarized state [55], the alpha dropout phenotype is consistent with individuals possessing a high latency between changes in thalamic and cortical SOP activity.

Overall, we see the following scenario playing out during the SOP: As a subject falls asleep, alpha diminishes. At some time following alpha dropout, there is an increase in delta and theta activity. During this transition period [12], [26], [32], the rapid tradeoff between the alpha and delta/theta power may occur. The latency between the loss of alpha, and the rise of delta/theta, is highly dependent on the individual. Behavioral responses may persist after loss of alpha, but they cease completely in the presence of strong delta/theta power. Therefore, we infer that *both* the absence of alpha and the presence of delta/theta are necessary for loss of behavioral response. This conclusion is supported by studies relating high theta power to poor PVT task performance [56].

Given this scenario, it follows that a subject's individual behavioral and physiological SOP phenotype is likely due to natural or pathological variation the intrinsic factors governing the sophisticated interplay of multiple thalamocortical systems. Our model provides a framework for explicitly modeling the dynamics of observations related to this interplay. It may therefore provide an essential non-invasive tool for the characterization of the state of the systems underlying SOP, as well as a means of quantifying the factors responsible for various phenotypes.

### The Wake Probability Framework as a Platform for Further Study of SOP Dynamics

It is clear that alpha, delta, and theta are not the only oscillations in play during the SOP, nor are they spatially static. Fortunately, our framework provides a straightforward means of implementing more sophisticated models of wakefulness. Future models can incorporate additional physiological observations such as slow (<1 Hz), beta (15–30 Hz), sigma (12–15 Hz) and gamma (>30 Hz) power, EEG spatial and coherence dynamics, and other biomarkers of sleep such as body temperature, heart rate, blood pressure, and more. The model may also be augmented to include other behavioral measures.

This model flexibility provides several major benefits. By adding more observation modalities, we can develop a model that fully captures our current understanding of the multiple systems affected during the SOP. Furthermore, continued model adjustments will allow SOP analysis to keep pace with new findings. Finally, since the behavioral component of this framework can be adjusted to characterize any other task or removed to account for no behavioral data at all, we can therefore easily apply this analysis to data previously collected in other experiments.

### Practical Applications of Wake Probability Analysis

Future work will focus on the many practical applications of our methods. Using our statistical framework, we can build models to rigorously characterize and compare the SOP phenotypes of different clinical populations, as well as to continue to characterize the natural heterogeneity of healthy subjects. By relating model component temporal dynamics to known linkages between observations and their underlying neural systems, this sort of analysis may help to shed further light on the pathophysiology of sleep. Furthermore, we can use our likelihood analysis to assess the relative goodness-of-fit of any set of proposed models, determining which model best fits the data. In doing so, we can provide a means of assigning any newly observed data to the phenotype or pathology associated with the model with the maximum likelihood, thus creating an efficient and principled means of automated diagnosis or categorization.

Additionally, by characterizing the time course of the wake probability curve itself, we can quantify differences in the rapidity of sleep onset in a principled manner. This analysis could act as a diagnostic tool for disorders of sleep onset, by comparing a subject's wake probability curve to those from population possessing a known pathology. If we adapt techniques for analyzing group behavior [57], the time course for sets of wake probability curves could also be compared, providing a way to analyze the influence of factors such as pharmacological agents, pathology, and the first night effect on the SOP.

Furthermore, wake probability could be used to dynamically track drowsiness in situations in which alertness is vital. Rather than attempt to detect the onset of sleep, it may be more important to detect the point at which wakefulness and the behavior associated with it decline.

## Materials and Methods

### Ethics Statement

Human studies were approved by the Human Research Committee of Massachusetts General Hospital, Boston, MA.

### Subjects

Ten healthy right-handed subjects (5 men and 5 women) with ages ranging 19–32 years (mean: 25.8, std: 5.09) and BMI <30 slept for two consecutive nights in our sleep laboratory. Subjects were screened to ensure a regular sleep schedule and no history of sleep disorder, psychiatric problem, or neurological disease, as well as to ensure no history of tobacco, or prescription/recreational drug use. We performed one night of home monitoring to exclude obstructive sleep apnea (OSA) screening (using a threshold of AHI <5, and RDI <15) (WatchPAT, Itamar Medical). A trained technician analyzed the experimental PSG data following the first experimental night, and one subject was excluded after failing to meet the OSA criteria on the first night). Urine tests for drug use (Xalex Multi Drug Kit for 10 Drugs) occurred at screening and prior to each experimental night. Female subjects were also screened for pregnancy.

### Experimental Recording and Data Processing

Subjects were fit with a high-density (64-channel) EEG cap, as well as standard clinical PSG sensors including PTAF, airflow, abdominal belt, and eye, chin, and limb electrodes.

EMG data were bandpass filtered between 10 and 70 Hz with the addition of a notch filter at 60 Hz. Airflow and abdominal belt data were bandpass filtered between .1 and 12 Hz. EEG and DC channel data were unfiltered. Multitaper spectrograms of the EEG data from 8 occipital channels were computed with 6 s temporal windows and 0.25 s overlap, a time-bandwidth product of 3, and 5 tapers. Delta, theta, and alpha power were defined as the total multitaper spectral power between 0.5–5 Hz, 5–8 Hz, and 8–12 Hz, respectively, of the median of the 8 occipital EEG channel spectrograms. It should be noted that the frequency band definitions for alpha, delta, and theta bands are not universally standardized, and thus vary between subfields within in the electroencephalography literature.

Visual staging of sleep data was performed prior to the statistical analysis by an experienced clinical sleep technician using contemporary AASM guidelines [5], [58].

### Formulation of the Wake Probability Model of the Sleep Onset Process

#### Overview

To better characterize the dynamics of falling asleep, we use a state-space framework [6], [42]–[44] to model the SOP in terms of a set of state variables that underlie our experimental data observations. We wish to integrate information from simultaneous measurements of task-related behavior, EMG, occipital EEG alpha power, and EEG delta/theta power to characterize the SOP. We begin by defining the random variables *x^m^*, *x^α^*, and *x* ^Δ*θ*^, representing the activity in the systems underlying our observations of EMG, occipital alpha oscillation power, and delta/theta oscillation power, respectively. We model these state processes such that they each represent the underlying activity level of the related neural system given the corresponding EEG/EMG observations. We then use the combined activity levels from the three systems along with the behavioral task responses to compute the probability that the subject is awake at each point in time. All model parameters and states are fit simultaneously from the experimental data with a particle filter. The resulting wake probability curve provides a continuous metric of wakefulness that tracks and characterizes SOP dynamics.

The analysis is performed in discrete time, where Δ*t* is the time interval between each of the *T* observations *t* = {1,…,*T*}.

### State Models

We first define the *state equations*, which model the temporal evolution of the state processes 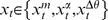 over time.

We define the random walk process at time *t* as
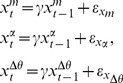
(1)where 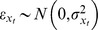 and 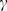 is a constant.

#### Probability of wake state

We define 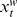, the state process related to waking at t, as a linear combination of the motor, alpha, and delta-theta states
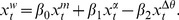
(2)where *β* = 1/3, so that 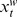 is the mean of the observation state distributions and
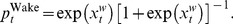
(3)


### Observation Models

#### EMG squeeze amplitude

We define *m_t_* as the log of the experimentally recorded EMG squeeze amplitude at time *t*, where
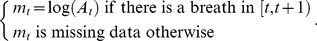
(4)


We then set up a first order linear relationship between the state and the log of the squeeze amplitudes, such that
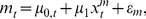
(5)where 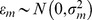, *μ*_1_ is a model coefficient, and 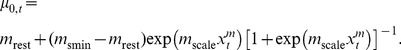
(6)


#### EEG power

We define a the EEG observation model as a sigmoidal function of the state, such that
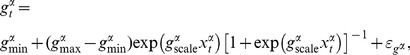
(7)for the alpha power process, and
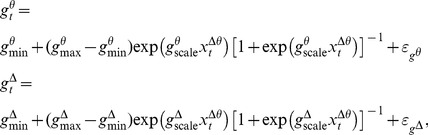
(8)


#### Behavioral binary responses

We define *b_t_* as the binary response at time *t*, where
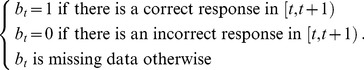
(9)


We model the Pr(*b_t_*), the probability of the behavioral response at *t*, as a binomial
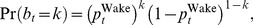
(10)


### Model Coefficients

To estimate the value of the model coefficients that are not time-varying 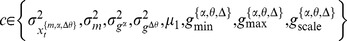, we used a random walk 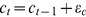
(11)where 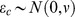 and 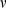 is small. This leaves room for some exploration of the parameter space without allowing for any large changes in the parameter value.

The coefficients and priors used in this model can be found in the *Supplementary Materials, Implementation Details*.

### Likelihood

Given our state and observation models, we can construct *θ_t_*, a vector of the parameter values at *t* such that
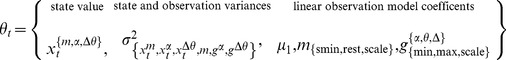
(12)


For each *θ_t_*, we can estimate the posterior density 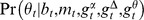
—the probability of all the model parameters *θ_t_* given the data. The posterior density is proportional tolog(*L*(*θ_t_*)), the joint log-likelihood of all the observations given the parameters.

We sum the log-likelihoods of all the observation processes to construct log(*L*(*θ_t_*)). Given the binomial loglikelihood for the binary responses
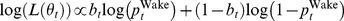
(13)and using Gaussian likelihoods for the continuous-valued observations, the full log-likelihood becomeswhere *I_b,t_*, *I_m,t_*, and 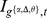 are indicator functions for each type of observation at time *t*. These indicator functions take on the value of 1 if the corresponding observation is present and 0 if it is missing. This sets up a flexible likelihood function that is able to deal with missing data for any of the observations. Furthermore, any time there is missing data for any reason (such as a disconnected EEG or a faulty connection), the log-likelihood can be constructed from whatever remaining data is available.

### Computing SOP Population Spectrograms

Given time-frequency observations from EEG data during the SOP, from *S* subjects, over discrete times 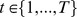, and fixed-width frequency bins centered at frequencies 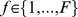, we define a matrix *Y^s^* as
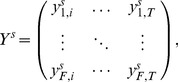
(15)such that 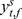 is the magnitude of the power spectrum for subject *s* at time *t* within the frequency bin *f*.

We also divide wake probability space into discrete bins 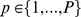, which divide the interval [0, 1] into *P* non-overlapping bins.

We define the SOP population spectrogram Φ(*p*, *f*), as:
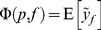
(16)where 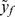 is the subset of all the 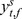 for all subjects in which 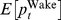 falls within bin *p*. In all cases, the median may be substituted for the expectation.

## Supporting Information

Code S1 Matlab code for estimating instantaneous wake probability from simultaneously observed EEG, EMG, and behavioral data.
(M) Click here for additional data file.

Figure S1 Data from the first (A) and second (B) consecutive experimental night for a subject with the alpha dropout phenotype. In this subject, for both nights, alpha diminishes before loss of behavioral response.
(EPS)

Figure S2 Data from the first (A) and second (B) consecutive experimental night for a subject with the alpha dropout phenotype. In this subject, for both nights, alpha diminishes before loss of behavioral response.
(EPS)

Protocol S1 Technical details on the model implementation, the particle filter algorithm, and the Bayesian goodness-of-fit procedure.
(DOCX)

## References

[1] Ogilvie RD (2001) The process of falling asleep. Sleep Med Rev 5: 247–270 10.1053/smrv.2001.0145 12530990

[2] Léger D, Bayon V (2010) Societal costs of insomnia. Sleep Med Rev 14: 379–389 10.1016/j.smrv.2010.01.003 20359916

[3] Vgontzas AN, Fernandez-Mendoza J, Liao D, Bixler EO (2013) Insomnia with objective short sleep duration: the most biologically severe phenotype of the disorder. Sleep Med Rev 17: 241–254 10.1016/j.smrv.2012.09.005 23419741 PMC3672328

[4] Richardson GS, Carskadon MA, Flagg W (1978) Excessive daytime sleepiness in man: multiple sleep latency measurement in narcoleptic and control subjects. Electroencephalography … 45: 621–627 10.1016/0013-4694(78)90162-1 PMC2391074 81764

[5] Iber (2007) The AASM Manual for the Scoring of Sleep and Associated Events: Rules, Terminology and Technical Specification.

[6] Smoothness Priors in Time Series (1987) Smoothness Priors in Time Series: 53. Available: http://books.google.com/books?id=4ndbGwAACAAJ&dq=kitagawa+gersch&hl=&cd=26&source=gbs_api.

[7] Badia P, Harsh J, Culpepper J, Shaffer J (1988) Behavioral control of abnormal breathing in sleep. J Behav Med 11: 585–592 10.1007/BF00844907 3252050

[8] Badia P, Harsh J, Balkin T, Cantrell P, Klempert A, et al (1984) Behavioral Control of Respiration in Sleep. Psychophysiology 21: 494–500 10.1111/j.1469-8986.1984.tb00231.x 6473618

[9] Shahid A, Wilkinson K, Marcu S, Shapiro CM (2012) STOP, THAT and One Hundred Other Sleep Scales. Springer. 1 pp.

[10] Pardey J, Roberts S, Tarassenko L, Stradling J (1996) A new approach to the analysis of the human sleep/wakefulness continuum. J Sleep Res 5: 201–210. 9065871 10.1111/j.1365-2869.1996.00201.x

[11] Hoddes E, Zarcone V, Smythe H, Phillips R, Dement WC (1973) Quantification of Sleepiness: A New Approach. Psychophysiology 10: 431–436 10.1111/j.1469-8986.1973.tb00801.x 4719486

[12] Ogilvie RD, Wilkinson RT (1984) The detection of sleep onset: behavioral and physiological convergence. Psychophysiology 21: 510–520. 6473620 10.1111/j.1469-8986.1984.tb00234.x

[13] Ogilvie RD, Wilkinson RT, Allison S (1989) The detection of sleep onset: behavioral, physiological, and subjective convergence. Sleep 12: 458–474. 2799219 10.1093/sleep/12.5.458

[14] Ogilvie RD, Wilkinson RT (1988) Behavioral versus EEG-based monitoring of all-night sleep/wake patterns. Sleep 11: 139–155. 3381055 10.1093/sleep/11.2.139

[15] MacLean AW, Arnedt T, Biedermann H, Knowles JB (1992) Behavioural responding as a measure of sleep quality. Sleep Research 21: 105.

[16] Balkin TJ (2011) Behavioral biomarkers of sleepiness. J Clin Sleep Med 7: S12–S15 10.5664/JCSM.1344 22003322 PMC3190422

[17] Marino M, Li Y, Rueschman MN, Winkelman JW, Ellenbogen JM, Solet JM, Dulin H, Berkman LF, Buxton OM (2013) Measuring sleep: accuracy, sensitivity, and specificity of wrist actigraphy compared to polysomnography. Sleep 36: 1747–1755 10.5665/sleep.3142 24179309 PMC3792393

[18] Buxton OM, Ellenbogen JM, Wang W, Carballeira A, O'Connor S, et al (2012) Sleep disruption due to hospital noises: a prospective evaluation. Ann Intern Med 157: 170–179 10.7326/0003-4819-157-3-201208070-00472 22868834

[19] Casagrande M, De Gennaro L, Violani C, Braibanti P, Bertini M (1997) A finger-tapping task and a reaction time task as behavioral measures of the transition from wakefulness to sleep: which task interferes less with the sleep onset process. Sleep 20: 301–312. 9231957 10.1093/sleep/20.4.301

[20] Hauri PJ, Wisbey J (1992) Wrist actigraphy in insomnia. Sleep 15: 293–301. 1519002 10.1093/sleep/15.4.293

[21] Kelly JM, Strecker RE, Bianchi MT (2012) Recent developments in home sleep-monitoring devices. ISRN Neurol 2012: 768794 10.5402/2012/768794 23097718 PMC3477711

[22] Blake H, Gerard RW, Kleitman N (1939) Factors influencing brain potentials during sleep. J Neurophysiol.

[23] De Gennaro L, Ferrara M, Bertini M (2001) The boundary between wakefulness and sleep: quantitative electroencephalographic changes during the sleep onset period. Neuroscience 107: 1–11. 10.1016/s0306-4522(01)00309-8 11744241

[24] Rechtschaffen A, Kales A (1968) A Manual of Standardized Terminology, Techniques, and Scoring Systems for Sleep Stages of Human Subjects. (1968). doi:10.1234/12345678.

[25] Hori T, Hayashi M, Morikawa T (1994) Topographical EEG changes and the hypnagogic experience. Washington, DC, US: American Psychological Association. 17 pp. doi:10.1037/10166-014.

[26] Davis H, Davis PA, Loomis AL, Harvey EN, Hobart G (1937) Changes in human brain potentials during the onset of sleep. Available: http://psycnet.apa.org/psycinfo/1938-00632-001. 10.1126/science.86.2237.448 17838964

[27] Magnin M, Rey M, Bastuji H, Guillemant P, Mauguière F, et al (2010) Thalamic deactivation at sleep onset precedes that of the cerebral cortex in humans. Proc Natl Acad Sci USA 107: 3829–3833 10.1073/pnas.0909710107 20142493 PMC2840430

[28] Nir Y, Staba RJ, Andrillon T, Vyazovskiy VV, Cirelli C, et al (2011) Regional slow waves and spindles in human sleep. Neuron 70: 153–169 10.1016/j.neuron.2011.02.043 21482364 PMC3108825

[29] Vyazovskiy VV, Olcese U, Hanlon EC, Nir Y, Cirelli C, et al (2011) Local sleep in awake rats. Nature 472: 443–447 10.1038/nature10009 21525926 PMC3085007

[30] Nobili L, De Gennaro L, Proserpio P, Moroni F, Sarasso S, et al (2012) Local aspects of sleep: observations from intracerebral recordings in humans. Prog Brain Res 199: 219–232 10.1016/B978-0-444-59427-3.00013-7 22877668

[31] Tanaka H, Hayashi M, Hori T (1997) Topographical characteristics and principal component structure of the hypnagogic EEG. Sleep 20: 523–534. 9322268 10.1093/sleep/20.7.523

[32] Marzano C, Moroni F, Gorgoni M, Nobili L, Ferrara M, et al (2013) How we fall asleep: regional and temporal differences in electroencephalographic synchronization at sleep onset. Sleep Med 14: 1112–1122 10.1016/j.sleep.2013.05.021 24051119

[33] Werth E, Achermann P, Borbély AA (1997) Fronto-occipital EEG power gradients in human sleep. J Sleep Res 6: 102–112. 9377529 10.1046/j.1365-2869.1997.d01-36.x

[34] Tinguely G, Finelli LA, Landolt H-P, Borbély AA, Achermann P (2006) Functional EEG topography in sleep and waking: state-dependent and state-independent features. NeuroImage 32: 283–292 10.1016/j.neuroimage.2006.03.017 16650779

[35] De Gennaro L, Ferrara M, Curcio G, Cristiani R (2001) Antero-posterior EEG changes during the wakefulness–sleep transition. Clinical neurophysiology 112: 1901–1911 10.1016/S1388-2457(01)00649-6 11595150

[36] Santamaria J, Chiappa KH (1987) The EEG of drowsiness in normal adults. J Clin Neurophysiol 4: 327–382. 3316272 10.1097/00004691-198710000-00002

[37] Tanaka H, Hayashi M, Hori T (1996) Statistical features of hypnagogic EEG measured by a new scoring system. Sleep 19: 731–738. 9122561 10.1093/sleep/19.9.731

[38] Alloway CE, Ogilvie RD, Shapiro CM (1999) EEG spectral analysis of the sleep-onset period in narcoleptics and normal sleepers. Sleep 22: 191–203. 10201063 10.1093/sleep/22.2.191

[39] Lamarche CH, Ogilvie RD (1997) Electrophysiological changes during the sleep onset period of psychophysiological insomniacs, psychiatric insomniacs, and normal sleepers. Sleep: Journal of Sleep Research & …. 9406324

[40] Cervena K, Espa F, Perogamvros L, Perrig S, Merica H, et al.. (2013) Spectral analysis of the sleep onset period in primary insomnia. Clin Neurophysiol. doi:10.1016/j.clinph.2013.10.010. 10.1016/j.clinph.2013.10.010 24239455

[41] Rechtschaffen A, Hauri P, Zeitlin M (1966) Auditory awakening thresholds in REM and NREM sleep stages. Percept Mot Skills 22: 927–942. 5963124 10.2466/pms.1966.22.3.927

[42] Barber D, Cemgil AT, Chiappa S (2011) Bayesian time series models.

[43] Durbin J, Koopman SJ (2001) Time series analysis by state space methods Oxford University Press. New York.

[44] Kim C-J, Nelson CR (1999) State-Space Models with Regime Switching: Classical and Gibbs-Sampling Approaches with Applications. MIT Press Books 1.

[45] Efron B, Tibshirani R (1993) An introduction to the bootstrap. New York: Chapman & Hall.

[46] Efron B, Gong G (1983) A leisurely look at the bootstrap, the jackknife, and cross-validation. The American Statistician 37: 36–48 10.1080/00031305.1983.10483087

[47] Dang-Vu TT, McKinney SM, Buxton OM, Solet JM, Ellenbogen JM (2010) Spontaneous brain rhythms predict sleep stability in the face of noise. Curr Biol 20: R626–R627 10.1016/j.cub.2010.06.032 20692606

[48] Clark ME, Hirschman R (1990) Effects of paced respiration on anxiety reduction in a clinical population. Biofeedback Self Regul 15: 273–284 10.1007/BF01011109 2223892

[49] Pramanik T, Sharma HO, Mishra S, Mishra A, Prajapati R, et al (2009) Immediate effect of slow pace bhastrika pranayama on blood pressure and heart rate. J Altern Complement Med 15: 293–295 10.1089/acm.2008.0440 19249921

[50] Sargunaraj D, Lehrer PM, Hochron SM, Rausch L, Edelberg R, et al (1996) Cardiac rhythm effects of.125-Hz paced breathing through a resistive load: implications for paced breathing therapy and the polyvagal theory. Biofeedback Self Regul 21: 131–147. 8805963 10.1007/BF02284692

[51] Telles S, Desiraju T (1991) Oxygen consumption during pranayamic type of very slow-rate breathing. Indian J Med Res 94: 357–363. 1794892

[52] Stancák A, Pfeffer D, Hrudová L, Sovka P, Dostálek C (1993) Electroencephalographic correlates of paced breathing. Neuroreport 4: 723–726 10.4187/respcare.02570 8347815

[53] Prerau MJ, Smith AC, Eden UT, Yanike M, Suzuki WA, et al (2008) A mixed filter algorithm for cognitive state estimation from simultaneously recorded continuous and binary measures of performance. Biol Cybern 99: 1–14 10.1007/s00422-008-0227-z 18438683 PMC2707852

[54] Prerau MJ, Eden UT (2011) A general likelihood framework for characterizing the time course of neural activity. Neural Comput 23: 2537–2566 10.1162/NECOa00185 21732865

[55] Hughes SW, Crunelli V (2005) Thalamic mechanisms of EEG alpha rhythms and their pathological implications. Neuroscientist 11: 357–372 10.1177/1073858405277450 16061522

[56] Hoedlmoser K, Griessenberger H (2011) Event-related activity and phase locking during a psychomotor vigilance task over the course of sleep deprivation. Journal of sleep …. doi:10.1111/j.1365-2869.2010.00892.x. 10.1111/j.1365-2869.2010.00892.x PMC3439125 20977513

[57] Smith AC, Stefani MR, Moghaddam B, Brown EN (2005) Analysis and design of behavioral experiments to characterize population learning. J Neurophysiol 93: 1776–1792 10.1152/jn.00765.2004 15456798

[58] Silber MH, Ancoli-Israel S, Bonnet MH, Chokroverty S, Grigg-Damberger MM, et al (2007) The visual scoring of sleep in adults. J Clin Sleep Med 3: 121–131. 17557422

[59] Prerau MJ, Smith AC, Eden UT, Kubota Y, Yanike M, et al (2009) Characterizing learning by simultaneous analysis of continuous and binary measures of performance. J Neurophysiol 102: 3060–3072 10.1152/jn.91251.2008 19692505 PMC2777819

[60] Smith AC, Frank LM, Wirth S, Yanike M, Hu D, et al (2004) Dynamic analysis of learning in behavioral experiments. J Neurosci 24: 447–461 10.1523/JNEUROSCI.2908-03.2004 14724243 PMC6729979

