# Supplementary material for: Tracking the Sleep Onset Process: An Empirical Model of Behavioral and Physiological Dynamics
Source: PLoS Comput Biol. 2014 Oct 2;10(10):e1003866. doi: 10.1371/journal.pcbi.1003866 (PMC4183428; doi:10.1371/journal.pcbi.1003866)
Supplement: Protocol S1 — Technical details on the model implementation, the particle filter algorithm, and the Bayesian goodness-of-fit procedure. (DOCX) [file pcbi.1003866.s004.docx]

# Supplementary Materials

## Implementation Details

Below is a table of prior distributions and parameter values used in this implementation:

| Parameter Description | Distribution/Value | |
| --- | --- | --- |
| Time resolution | $\Delta t=0.25s$ | |
| Number of particles | $P=10000$ | |
| State value prior | $x_{0}^{m},x_{0}^{\alpha}\sim U\left(0,3\right), x_{0}^{\Delta\theta}\sim U\left(-3,0\right)$ | |
| State variance prior | $\sigma_{x,0}^{2}\sim U\left(\rho,0.5\right)$ | |
| EMG observation parameter priors | $\mu_{1}\sim U\left(0,1\right)$ $m_{\text{smin,rest}},\sim N\left(\rho,0.5\right)$, where$\rho$ is the 2.5 percentile of the $m$ when $b_{k}=1$ and $b_{k}=0$, respectively . | |
| EMG observation noise prior | $\sigma_{\mu,0}^{2}\sim U\left(0,.001\right)$ | |
| EEG Observation Parameter Priors | $g_{\min,\max}^{\left\{\alpha,\Delta\theta\right\}},\sim N\left(\rho,0.5\right)$, where$\rho$ is the 2.5 and 97.5 percentile of the power of $g^{f}$, respectively. $g_{\text{scale}}^{\left\{\alpha,\Delta\theta\right\}}\sim U\left(0,0.5\right)$ | |
| EEG Observation Noise Prior | $\sigma_{g^{\left\{\alpha,\Delta\theta\right\}}}^{2}=U\left(0,10\right)$ | |
| Random Walk Parameter | $\gamma=.9999$ | |
| Coefficient variances | Parameter(s) | Value of $\nu$ |
|  | $\text{ }\varepsilon_{\sigma_{x_{k}^{\left\{\alpha,\Delta\theta\right\}}}^{2}},m_{\left\{\min,\max,\text{scale}\right\}},g_{\left\{\min,\max,\text{scale}\right\}}^{\left\{\alpha,\Delta\theta\right\}}$ | 0.01 |
|  | $\varepsilon_{\sigma_{x_{k}^{m}}^{2}}$ | 0.02 |
|  | $\varepsilon_{g^{\left\{\alpha,\Delta\theta\right\}}}$ | 0.1 |
|  | $\varepsilon_{\sigma_{m}^{2}}$ | 0.0002 |
|  | $\mu_{1}$ | 0.004 |
| Parameter Description | Distribution/Value | |
| Time resolution |  | |

## Particle Filter

To estimate $\theta_{t}$ at each time, we construct a particle filter, which is an algorithm based on a Bayesian resampling procedure. The idea of a particle filter is to create a large set of parameter vectors (called particles) with different realizations of $\theta_{t}$ that evolve over time based on the model equations and observations. The particle filter algorithm is designed such that the distribution of these parameter vectors is an approximation of the posterior density.

To implement the particle filter, the initial values for each particle are first drawn from a proposal density $\pi\left(\theta_{1}\right)$, also known as a prior, which is our best guess of the distributions of initial conditions of for each element of $\theta_{t}$. Given a set of particles drawn from the proposal density, the time-varying elements in each particle are advanced through the state and parameter one-step prediction equations. If observations are present, the likelihood of each particle given the data is computed and used as a weight to determine the probability with which the particle will be resampled at the next time step. In this way, the distribution of the weights acts as $\pi\left(\theta_{t+1}\right)$, the proposal density for the next time step. The particles are then resampled with replacement according to the new proposal density, and the process is repeated again for all subsequent time steps.

The iterative procedure is as follows:

### Particle Filter Algorithm

Given a set of $P$ particles, where $\rho_{t}^{i}$is the $i$^th^ particle at $t$, is a realization of the model parameters $\theta_{t}$:

1. Define $\pi\left(\theta_{0}\right)$, the prior distribution the parameters to be estimated
2. For each time $t=\left\{1,...,T\right\}$, and for all particles $\left\{\rho_{t}^{1},...,\rho_{t}^{P}\right\}$
   1. Sample with replacement from the proposal density such that $\rho_{t}^{i}\sim\pi\left(\theta_{t-1}\right)$ for $i\in\left\{1,...,P\right\}$
   2. Update the state and state variance parameters using (1).
   3. Compute a weight vector $w^{i}$for each particle such that$w^{i}=L\left(\rho_{t|t-1}^{i}\right)$, where $\rho_{t|t-1}^{i}$is the particle value after the state update, and $L\left(\rho_{t|t-1}^{i}\right)$ is the likelihood of the particle given the observations, computed by exponentiating the loglikelihood (16).
   4. Compute $\pi\left(\theta_{t}\right)$, the posterior distribution estimate at time $t$ by normalizing the weights such that $\sum_{i=1}^{P} w^{i}=1$.
3. The particles $\left\{\rho_{t}^{1},...,\rho_{t}^{P}\right\}$ act as an estimate of the time-varying posterior distribution of $\hat{\theta}_{t}$.
4. The distribution median and confidence intervals with significance $\alpha$can be computed using the component-wise 50^th^,$\alpha/2$ and $1-\alpha/2$ percentiles of $\left\{\rho_{t}^{1},...,\rho_{t}^{P}\right\}$, respectively.
5. The *wake probability curve* is defined as $\hat{\theta}_{1...T}$the posterior distribution across the entire sleep onset period, and is visualized using the median and 95% confidence bounds of the particles.

## Bayesian Goodness-of-Fit Analysis

### Computing the Total Loglikelihood Distribution

In order to compare the wake probability model to the binary switch models, we devised a Bayesian goodness-of-fit analysis to determine how well each model class estimates the observed behavioral response data across all of the subjects. As each behavioral task response is binary (correct = 1, incorrect = 0), we can view the responses as Bernoulli trials. Given the behavioral data and the estimated probability of response from a specific model $m$, we can therefore compute $\log\left(L\right)$ the binomial loglikelihood of the data given the model as

$\log\left(L\right)=b_{t}\log\left(p_{m,t}\right)+\left(1-b_{t}\right)\log\left(1-p_{m,t}\right)$,

where at time $t$, $b_{t}$ is the behavioral observation, and $p_{m,t}$ is the estimated response probability from model $m$, assuming that $\Pr\left(\text{Wake}\right)\propto\Pr\left(\text{Response}\right)$.

Computed across all time for all subjects, we get the total loglikelihood

$\log\left(L_{\text{total}}\left(p_{m}\right)\right)=\sum^{\text{subjects}} \sum_{t=1}^{T} b_{t}\log\left(p_{m,t}\right)+\left(1-b_{t}\right)\log\left(1-p_{m,t}\right)$.

In a Bayesian framework, we estimate the posterior density of response/wake probability of $p_{m,t}$, which the distribution $f\left(p_{m,t}|\text{data}\right)$, rather than a constant. Consequently, the loglikelihood will also be a distribution.

In order to make a comparison between two models $m_{1}$ and $m_{2}$ we can estimate the difference distribution of the respective total loglikelihoods as

$\log\left(L_{\text{total}}\left(p_{m_{1}}\right)-L_{\text{total}}\left(p_{m_{2}}\right)\right)=\sum^{\text{subjects}} \sum_{t=1}^{T} b_{t}\log\left(p_{m_{1},t}-p_{m_{2},t}\right)+\left(1-b_{t}\right)\log\left(1-\left(p_{m_{1},t}-p_{m_{2},t}\right)\right)$.

The Bayesian credible interval with which the total loglikelihood of $m_{1}$ is greater than that of $m_{2}$is the proportion of $\log\left(L_{\text{total}}\left(p_{m_{1}}\right)-L_{\text{total}}\left(p_{m_{2}}\right)\right)$ that rests above 0.

### A Bayesian Framework for Instantaneous transition Models

For the wake probability model, $f\left(p_{m,t}|\text{data}\right)=\Pr(Wake)$, which is approximated by the particles from the particle filter.

The instantaneous transition models, however, classify sleep stage data from the hypnogram into an absolute determination of sleep or wake. To perform a comparison with the wake probability model, we must place the instantaneous transition models into a Bayesian framework

[61] by computing the posterior distributions for the each model. We make the assumption that a waking subject will perform the behavioral task correctly with significance (95 correct responses out of 100 trials), and that a sleeping subject will perform incorrectly with significance (5 correct responses out of 100 trials).

In the context of a Bernoulli experiment with trials with correct responses, we recall from Bayes’ law that

$\overbrace{f\left(p_{m,t}|k\right)}^{\text{posterior}}\propto\overbrace{f\left(k|p_{m,t}\right)}^{\text{likelihood}}\overbrace{f\left(p_{m,t}\right)}^{\text{prior}}$.

For the likelihood, we use the binomial probability model

$f\left(k|p_{m,t}\right)=\left(\begin{matrix} n \\ k \end{matrix}\right)p_{m,t}^{k}\left(1-p_{m,t}\right)^{n-k}$.

We model the prior density as a beta distribution

$f\left(p_{m,t}\right)\sim Beta\left(\alpha,\beta\right)=\frac{\Gamma\left(\alpha+\beta\right)}{\Gamma\left(\alpha\right)\Gamma\left(\beta\right)}p_{m,t}^{\alpha-1}\left(1-p_{m,t}\right)^{\beta-1}$ ,

where $\alpha>0$ and $\beta>0$, and $\Gamma$ is the gamma function $\Gamma\left(\alpha\right)=\int_{0}^{\infty} x^{\alpha-1}\exp\left(-x\right)dx$. We use $\alpha=1$ and $\beta=1$ to make the prior uniform and therefore uninformative.

We can then compute the posterior probability density as the product of the likelihood and the prior , which is

$f\left(p_{m,t}|k\right)=\frac{\Gamma\left(n+\alpha+\beta\right)}{\Gamma\left(k+\alpha\right)\Gamma\left(n-k+\beta\right)}p_{m,t}^{k+\alpha-1}\left(1-p_{m,t}\right)^{n-k+\beta-1}$.

By plugging $\alpha=\beta=1$ into , this simplifies to the beta distribution

$f\left(p_{m,t}|k\right)=Beta\left(k+1,n-k+1\right)$,

where

$\left\{\begin{matrix} n=100,k=95\text{ if the binary model indicates }Wake \\ n=100,k=5\text{ if the binary model indicates }Sleep \end{matrix}\right.$.

Thus, given an output of *Wake* or *Sleep* from any instantaneous transition model, we can compute the posterior distribution of the response probability given the data.

### Monte Carlo Estimation of Model Loglikelihood Distributions

We can estimate the loglikelihood distributions using a Bayesian Monte-Carlo simulation by repeatedly drawing values of $p_{m,t}$ from a given model’s posterior distribution, $f\left(p_{m,t}|\text{data}\right)$ and plugging into the loglikelihood equation . The normalized histogram of the results will approximate the distribution on the loglikelihood. To compute the loglikelihood difference distribution, the same procedure is followed using .

The Monte Carlo procedures are as follows:

#### Monte Carlo Estimation of the Total Loglikelihood Distribution

1. For each of $N$ samples:
   1. Compute $\log\left(L_{\text{total}}\left(p_{m}\right)\right)$ from
   2. If the model is the wake probability model, $p_{m,t}$ is drawn from the set of particles approximating the posterior distribution.
   3. If the model is a binary switch model, $p_{m,t}$ is drawn from the appropriate Beta distribution, given by and .
2. The loglikelihood distribution is estimated from the histogram of $\log\left(L_{\text{total}}\left(p_{m}\right)\right)$ from the samples
3. The Bayesian credible interval of $\alpha$ can be computed using the $\alpha/2$ and $1-\alpha/2$ sample percentiles

#### Monte Carlo Estimation of the Total Loglikelihood Difference Distribution and Bayesian Credible Interval

1. For each of $N$ samples:
   1. Compute $\log\left(L_{\text{total}}\left(p_{m_{1}}-p_{m_{2}}\right)\right)$ from
   2. If a model is the wake probability model, $p_{m,t}$ is drawn from the particles estimating the posterior distribution.
   3. If a model is a binary switch model, $p_{m,t}$ is drawn from the appropriate Beta distribution, given by and .
2. Compute the histogram of $\log\left(L_{\text{total}}\left(p_{m_{1}}-p_{m_{2}}\right)\right)$ from the samples
3. The Bayesian credible interval for is proportion of samples where $\log\left(L_{\text{total}}\left(p_{m_{1}}-p_{m_{2}}\right)\right)>0$

In our implementation, $N=10000$. We computed the total loglikelihood distribution across all subjects and nights for each model. We then computed the difference distribution between the wake probability model and each of the instantaneous transition models. As the wake probability model incorporates information the behavioral data, we used the posterior distribution from the time step prior to the behavioral observation in all of the goodness-of-fit analyses. This is equivalent to a leave-one-out cross-validation scheme, in which the data point to be estimated is removed from model fitting and prediction procedure.
